# Migration control enters the clinic: a public health red line

**DOI:** 10.1016/j.lanepe.2026.101703

**Published:** 2026-05-04

**Authors:** The Lancet Regional Health – Europe

Across Europe, health-care professionals are warning against an intensifying direction in migration policy. Following proposals under negotiation for the EU Return Regulation, aimed at expanding deportations and introducing broad detection measures, health-care workers have warned that these provisions could require them to identify or report undocumented individuals, effectively linking access to care with immigration enforcement. In response, more than 1100 health-care workers across Europe have stated that they will not become “instruments of immigration enforcement”, cautioning that such measures could deter people from seeking care and erode trust in the health-care profession.

This moment marks a crucial boundary for medical ethics and the integrity of health systems, at the point in which migration control enters the clinic. It represents a public health red line: when health care ceases to be a space of care and becomes an instrument of migration control. When health-care professionals are required to participate in enforcement or legitimise conditions known to harm health, core principles of medicine including confidentiality, trust, and the duty of care are undermined.

This tension is no longer theoretical and is already reshaping clinical practice. Recent correspondence in the journal highlights how clinicians are increasingly drawn into migration control processes. In Italy, infectious disease specialists have been investigated for issuing certificates of unfitness for detention to migrants held in pre-repatriation centers. This raises concerns about the criminalisation of clinical judgement and erosion of medical independence, and the risk of turning clinicians into notaries of the border, where medical practice is used to enforce administrative measures. In such contexts, medical decision-making directly contradicts the principle of *primum non nocere*—first, do no harm.

At European level, this policy shift is already taking shape. On March 26, 2026, the European Parliament voted to advance reforms to existing EU Return rules. The reforms aim to establish a more unified system for returning undocumented migrants, including the introduction of a European Return Order, expanded detention powers, and return hubs outside the EU where rejected asylum seekers could be detained before deportation. While presented as a measure to improve efficiency, this shift towards return-centred migration governance prioritises enforcement over health and reduces clinical judgement to administrative clearance for deportation.

The health consequences of immigration detention are well established. Detention is associated with increased risk of infectious disease, deterioration of chronic conditions, and a high prevalence of anxiety, depression, and post-traumatic stress disorder with longer detention linked to more severe outcomes. Detention conditions often prevent timely and appropriate treatment, and the integration of enforcement into health-care systems will further deter care-seeking and disrupt continuity of care.

Although international frameworks mandate that detention be used only as a last resort, its use is increasingly becoming the norm. The result is not only individual harm, but also wider public health consequences, including delayed diagnosis, fragmented care, greater transmission risk, increased strain on emergency services, and higher costs of national health systems having to deal with more complicated cases.

There is also a structural contradiction. European health systems are built on principles of universalism and equity, reflected in commitments to universal health coverage and the Sustainable Development Goals’ pledge to “leave no one behind”. Yet return-focused policies increasingly operate through exclusion, creating populations effectively excluded from health-care systems.

Migration policy is therefore not external to health systems, it is actively shaping them. Policies, including return and detention frameworks, should be subject to systematic and transparent health impact assessment. Safe and equitable access to health care must be guaranteed regardless of migration status and health-care professionals must not be placed in positions that compromise clinical independence or ethical obligations.

The refusal of health-care professionals to act as agents of immigration enforcement is, in effect, the drawing of a red line, but this boundary is unlikely to hold if migration governance continues to expand beyond the clinic. As a recent report has shown, universities and research institutions are increasingly embedded in the development and expansion of Europe's border regime, contributing data, technologies, and policy frameworks that reinforce deterrence-based approaches. If this trajectory continues, the red line between care and control will be crossed and redefined. Safeguarding the integrity of health-care systems now requires that red line to be defended.

